# Observational study: handgrip strength, body composition and diabetes mellitus

**DOI:** 10.1186/s13104-021-05731-4

**Published:** 2021-08-28

**Authors:** Naomi Suda, Chrispin Manda, Joshua Gallagher, Yukiko Wagatsuma

**Affiliations:** 1 School of Medicine and Medical Science, University of Tsukuba, 1-1-1 Tennodai, Tsukuba, Ibaraki 305-8575 Japan; 2 Department of Clinical Trials and Clinical Epidemiology, Graduate School of Comprehensive Human Sciences, University of Tsukuba, 1-1-1 Tennodai, Tsukuba, Ibaraki 305-8575 Japan; 3 Department of Public Health and Primary Care, School of Clinical Medicine, University of Cambridge, Cambridge, UK; 4 Department of Clinical Trials and Clinical Epidemiology, Faculty of Medicine, University of Tsukuba, 1-1-1 Tennodai, Tsukuba, Ibaraki 305-8575 Japan

**Keywords:** Diabetes, Handgrip strength, Body composition, Body mass index, Fat free mass

## Abstract

**Objective:**

Previous studies have shown that relative handgrip strength, handgrip measure divided by body mass index (BMI), affects the future onset of diabetes and prediabetes. However, fat free mass (FFM) has been suggested to adjust for this effect better than BMI. In this study, we examined applicability of models that adjusted handgrip-diabetes relationship with either BMI or FFM.

**Results:**

Of 1940 participants (56.2% male, average (SD) age, 57.2 [11.2] years), 267 (13.8%) had diabetes (DM) and 912 (47.0%) had prediabetes (pre-DM). The average handgrip measure for men was 40.0 kg (tertile measures, 37.4 kg and 42.5 kg) and for women 24.2 kg (tertile measures, 22.6 kg and 25.7 kg). Among both sexes, the percentage of people unaffected by DM or pre-DM was highest in the strong handgrip group and lowest in the weak handgrip group. Analysis using binary logistic models showed that an increase in handgrip measure was associated with a decrease in the chance of having either pre-DM or DM. This effect was detected by both BMI models and FFM models, even after adjustment for medical and lifestyle factors. Either or both should be used depending on the research aims, setting and methods.

**Supplementary Information:**

The online version contains supplementary material available at 10.1186/s13104-021-05731-4.

## Introduction

Diabetes Mellitus (DM) is a common lifestyle-related disease characterized by prolonged hyperglycemia due to impaired sugar metabolism. Although being obese is an established risk factor for developing DM [1], it is also common for low- and normal- BMI individuals to develop DM [2]. For this reason, body measures other than BMI should also be considered when assessing DM risk.

Handgrip strength is a simple measure of muscle strength adapted as a test for physical fitness. Handgrip strength has shown a significant correlation with other strength measures such as quadriceps strength [3]. Although the underlying mechanism has not been fully understood, studies on the effect of muscle resistance exercises to glucose metabolism reported that such muscle strengthening activities have effects on muscle function and glucose deposition [4]. One previous study has shown that relative handgrip strength (Handgrip/BMI) at baseline can predict the onset of DM within the next three years [5]. In addition, another study has shown that relative handgrip strength at baseline can also predict pre-DM [6]. A recent meta-analysis based on 10 observational cohort studies suggested that handgrip strength may be a risk indicator for type-2 diabetes [7]. In response to these findings, there have been suggestions that fat free mass (FFM) rather than BMI should be used to modify the handgrip strength. In this study, we compare BMI and FFM in terms of their ability to adjust the handgrip strength-diabetes relationship.

## Main text

### Materials and methods

#### Study area and population

This study was conducted in Mito City, Ibaraki prefecture under the Center of Innovation program of Japan, which aims to improve population health. Most of the study participants were members of the Japan Agriculture Cooperative of Ibaraki (JA Ibaraki). Participants either attended annual health checks organized in partnership with JA Ibaraki at the regional hospital (Mito-Kyodo Hospital) and its outreach service, or alternatively, health checks were organized by participants’ employers. The total number of annual attendances was 7391. The data was collected from April 2018 to November 2019.

We recruited individuals who were 20 to 70 years old and undertook both a body composition measurement and a handgrip strength test as part of their annual health check (N = 2167). Those whose diabetic status was not known according to the criteria described later were excluded (N = 227). The final number of participants was 1940.

#### Measurements and definitions

##### Physical measures

Anthropometric measures such as height, weight and fat free mass (FFM) were measured using a Tanita DC250 device (TANITA Co, Japan). Fasting blood samples were collected, with biomedical tests measuring hemoglobin A1c (HbA1c) and fasting plasma glucose (FPG) later being conducted at the regional laboratory. Body mass index (BMI; calculated as weight/height^2^) was used to define underweight (< 18.5 kg/m^2^), normal (18.5–24.9 kg/m^2^), or overweight-obese (≥ 25 kg/m^2^) participants.

##### Handgrip

We assessed handgrip strength using a Smedley digital handgrip test machine (Takei Corporation, Japan) following standard operating procedures. Participants were instructed to stand upright and look ahead as a dynamometer handle was placed in their palm. They were then told to grip the handle on their tested side, with the other arm positioned downwards and not touching their body or any other object.

##### DM status

DM was defined as having one of the following: use of antidiabetic medication; FPG above 126 g/dl; or HbA1c above 6.5%. Pre-DM was defined as having one of the following: FPG from 110 to 125 g/dl; or HbA1c of 5.7 to 6.4%. Non-DM was defined as fulfilling both criteria of a FPG under 110 g/dl and HbA1c under 5.7%. Individuals who did not fulfill criteria for any of the definitions were excluded.

##### Lifestyle and medical history

Participants answered questionnaires regarding their current medical treatment and lifestyle. Questions about the medical treatment were: “Are you using any anti-hypertensive drugs/anti-cholesteric agents/antidiabetic drugs or insulin injection?” (Yes or No). Regular physical activity was ascertained through the question: “Have you exercised more than twice a week for over a year, for at least 30 min per session?” (Yes or No). Participants smoking habit was then asked with a question: “Have you smoked in the last month?” (Yes or No).

#### Statistical analysis

Participants’ HbA1c measures, demographic-, anthropometric-, and lifestyle characteristics were reported as mean values with standard deviations (SD) or median values with interquartile ranges for continuous variables. Whole numbers and percentages were used for categorical variables.

Body composition (BMI, FFM and muscle mass) and handgrip strength were described as mean values and SDs, categorized by sex and age group (20 to 39, 40 to 59, and 60 years or older). We divided participants into three groups using sex-specific tertile scores of handgrip strength. Case numbers and percentages of each DM category (non-DM, pre-DM, and DM) within the three handgrip strength groups were reported. We performed a chi-square test to examine whether DM status and handgrip strength are related.

Finally, multivariable-adjusted logistic regression models were used to estimate the association between handgrip strength and having pre-DM or DM. We first employed models adjusted for age, sex and either BMI or FFM. We then examined models that included current medical treatment and lifestyle factors.

### Results

Age, sex, BMI, the number of individuals in each DM category (non-DM, pre-DM, DM) and lifestyle characteristics are described in Table 1. Males were 56.2% of the participants. The mean (SD) value for age was 57.2 years (11.2 years). Mean BMI (SD) of all participants was 23.7 kg/m^2^ (3.6 kg/m^2^).

**Table 1:** Participants’ demographic, medical and life-style characteristics

Characteristics	N = 1940
Age ± SD	57.2 ± 11.2
20–39 (%)	160 (8.2)
40–59 (%)	796 (41.0)
60–75 (%)	984 (50.7)
Sex—male %	56.2
BMI ± SD	23.7 ± 3.6
Status of diabetes mellitus	
DM (%)	267(13.8)
Pre-DM (%)	912 (47.0)
Non-DM (%)	761(39.2)
Treatment of diabetes mellitus	
Yes (%)	94 (4.9)
No (%)	1836 (95.1)
Treatment of hypertension	
Yes (%)	483 (24.9)
No (%)	1445 (74.5)
Treatment of dyslipidemia	
Yes (%)	357 (18.4)
No (%)	1573 (81.1)
Regular exercise	
Yes (%)	619 (31.9)
No (%)	1319 (68)
Current smoking	
Yes (%)	292 (15.1)
No (%)	1648 (84.9)

Participants’ body composition measures and handgrip strength are reported in the Additional file 1: Table S1. Women had lower BMI than men in all age groups, with the gap largest in the group of 40 to 59 years old. High BMI (≥ 25) was observed in 39.3% of males and 27.4% of females. Low BMI (< 18.5) was much more common in women (9.0%) than in men (1.9%). Mean values (SD) of handgrip strength were 40.0 kg (6.2 kg) for men and 24.2 kg (4.0 kg) for women, respectively. It is worth noting that while women’s handgrip strength and FFM decline relatively steadily by age, both scores for men drop sharply in the group of 60 years old or above.

In Table 2, we report the numbers and prevalence of non-DM, pre-DM and DM individuals in each handgrip strength tertile. The tertile values were 37.4 kg and 42.5 kg for men, and 22.6 kg and 25.7 kg for women. Although DM was more common among men (16.5%) than women (10.2%), the overall prevalence of pre-DM and DM combined was similar in both sexes (59.8% for men and 62.1% for women; p = 0.301 by chi-square test). Generally, there were more non-DM cases and less DM cases with each increase in handgrip strength group. Such a relationship was always true for women but not for the medium and strongest groups of men. Chi-square tests showed the relationship between handgrip strength and DM levels were significant among men (p = 0.022), women (p = 0.001) and both (p = 0.000).

**Table 2:** The number and percentage of DM by handgrip strength tertile

Handgrip tertile	Neither	Pre-DM	DM	ALL	p
Male					
Weak (%)	128 (34.7)	164 (44.4)	77 (20.9)	369 (100)	0.022
Medium (%)	154 (42.5)	159 (43.9)	49 (13.5)	362 (100)	
Strong (%)	157 (43.6)	149 (41.4)	54 (15.0)	360 (100)	
All (%)	439 (40.2)	472 (43.3)	180 (16.0)	1091 (100)	
Female					
Weak (%)	92 (32.2)	154 (53.8)	40 (14.0)	286 (100)	0.001
Medium (%)	100 (34.8)	158 (55.1)	29 (10.1)	287 (100)	
Strong (%)	130 (47.1)	128 (46.4)	18 (6.5)	276 (100)	
All (%)	322 (37.9)	440 (51.8)	87 (10.2)	849 (100)	
All					
Weak (%)	220 (33.6)	318 (48.5)	117 (17.9)	655 (100)	0.000
Medium (%)	254 (39.1)	317 (48.8)	78 (12.0)	649 (100)	
Strong (%)	287 (45.1)	277 (43.6)	72 (11.3)	636 (100)	
All (%)	761 (39.2)	912 (47.0)	267(13.8)	1940 (100)	

*Pre-DM* prediabetes, *DM* diabetes mellitus; p-values by chi-square test

The results from the analysis using multivariable-adjusted regression models of handgrip strength measure and pre-DM/DM prevalence are shown in Table 3. An increase in handgrip strength was associated with a decrease in the chance of having pre-DM or DM, after adjusting for age, FFM, and the interaction effect of FFM and sex (FFM model 1). This remained the case when adjusting the model for variables representing; treatment for dyslipidemia; treatment for hypertension; regular exercise and smoking (FFM model 2). A similar model with factors including handgrip strength, age, BMI and BMI-sex interaction is presented as BMI model 1, which also detected a similar size of effect. The addition of factors such as dyslipidemia treatment and smoking into the model did not change the result. Although both FFM and BMI models showed similar results, we observed notable differences when FFM model 1 and BMI model 1 were applied to different BMI groups (low, normal and high; Additional file 1: Table S2). When analysing the low-BMI group, only the FFM model detected a significant handgrip strength-DM relationship.

**Table 3:** Adjusted odds ratios of having DM or pre-DM for 5 kg increase in handgrip strength

Model	N	aOR (95% CI)
FFM model 1	1940	0.810 (0.729–0.899)
FFM model 2	1926	0.813 (0.731–0.905)
BMI model 1	1940	0.848 (0.708–0.937)
BMI model 2	1930	0.844 (0.764–0.932)

*aOR* adjusted odds ratio, *95% CI* 95% confidence interval, *FFM* at free mass, *BMI* body mass index

FFM model 1: Adjusted for age, FFM, and FFM*sex(interaction)

FFM model 2: Adjusted for age, FFM, FFM*sex(interaction), hypertension treatment, dyslipidemia treatment, exercise, and current tobacco smoking

BMI model 1: Adjusted for age, BMI, and BMI*sex(interaction)

BMI model 2: Adjusted for age, BMI, BMI*sex(interaction), dyslipidemia treatment, and current tobacco smoking

### Discussion

This study examined the cross-sectional relationship between handgrip strength and diabetes in a general population of Mito area of Japan. When comparing different models which predict current diabetes status based on handgrip strength, we found that FFM and BMI can adjust for this relationship equally well.

First, by comparing the prevalence of pre-DM and DM by handgrip tertile, we confirmed that in general, a stronger handgrip is linked with more non-DM cases and likewise less DM cases. A notable exception is that the strongest group of men had more DM cases than the medium strength group. This could be due to a high percentage of men with obesity in the strong handgrip group [8]. Indeed, obesity was much more prevalent in men compared to women in the overall groups (39.3% for men versus 27.4% for women) and in the strong-handgrip groups (49.7% for men versus 30.8% for women). Alternatively, this larger percentage of DM observed in the strongest group might be due to larger proportion of manual and physical laborers in this group. Lifestyle characteristics that are common in these workers, such as smoking, frequent alcohol consumption and an irregular circadian rhythm are considered risk factors for DM. In support of this hypothesis, one Australian study showed that blue-collar workers have a higher risk of type-2 diabetes compared to white-collar workers. [9]

Based on our multivariable-adjusted logistic regression analysis, we found that FFM can adjust for the effect of handgrip strength equally well when compared with BMI. The benefit of adjusting with FFM was notable when the models were applied to low- and high-BMI groups. BMI, which is acquired by dividing body mass (kg) with the square of height (m^2^) does not distinguish between body weight due to muscle or fat mass. FFM, on the other hand, is the absolute mass of bones, muscles, connective tissues and fluid within the body: hence it is affected by muscle mass and not fat mass. Furthermore, BMI is generally independent of height, while FFM is greatly affected by it. For these reasons, BMI and FFM measure different aspects of body composition and can be seen as complementary to each other. One study reported that persons with dynapenic obesity, characterized by high-BMI and low-FFM, are a subgroup of the population at particular risk of type-2 diabetes [10]. This supports the idea that BMI and FFM represent different aspects of one’s body composition.

When it comes to using handgrip strength as a risk factor of DM, our overall conclusion from this study is that BMI and FFM can adjust the handgrip strength-DM relationship equally well. Either or both should be used depending on the research aims, setting and methods.

## Limitations

The study population is limited in diversity in several ways. This study was conducted using the population in one area. Furthermore, because all participants were visitors of an annual health check, some of which are not mandatory and self-funded, this study may include a relatively health-conscious population. In this study, type-1 and type-2 diabetes were not separately considered. Although type-2 diabetes is much more common than type-1 diabetes, they should ideally not be conflated as they are different in their pathophysiology. Finally, there are few studies with results comparable with the present study's findings. This is a cross-sectional study that analyzes the relationship between handgrip strength and diabetes status. The relationship between these two variables should be further investigated in prospective and intervention studies. Additionally, a case–control design could bring about more reliable results.

## Supplementary Information

**Additional file 1: Table S1.** Participants’ body composition and handgrip strength by age group. **Table S2.** Adjusted odds ratios for having DM or pre-DM for 5kg increase in handgrip strength.

## Data Availability

The datasets used and/or analyzed during the study are available from the corresponding author on reasonable request.

## Abbreviations

BMI: Body mass index
DM: Diabetes mellitus
Pre-DM: Prediabetes
FFM: Fat free mass
FPG: Fasting plasma glucose
HA1c: Hemoglobin A1c
OR: Odds ratio
SD: Standard deviation
