# Supplementary material for: Observational study: handgrip strength, body composition and diabetes mellitus
Source: BMC Res Notes. 2021 Aug 28;14:332. doi: 10.1186/s13104-021-05731-4 (PMC8399788; doi:10.1186/s13104-021-05731-4)
Supplement: Supplementary file 1 — Additional file 1: Table S1. Participants’ body composition and handgrip strength by age group. Table S2. Adjusted odds ratios for having DM or pre-DM for 5kg increase in handgrip strength. [file 13104_2021_5731_MOESM1_ESM.docx]

**Additional Tables**

Table S1. Participants’ body composition and handgrip strength by age group

|  |  | BMI (kg/m^2^) | Absolute fat mass (kg) | Fat free mass (kg) | Muscle mass (kg) | Handgrip strength (kg) |
| --- | --- | --- | --- | --- | --- | --- |
| Men | Age -39 | 23.9±3.7 | 16.7±7.1 | 54.5±6.2 | 51.7±5.9 | 41.3±5.8 |
|  | Age 40-59 | 24.8±3.7 | 17.8±6.5 | 54.5±5.9 | 51.6±5.7 | 41.7±5.9 |
|  | Age 60- | 24.0±2.9 | 16.7±13.4 | 50.9±5.2 | 48.0±5.4 | 38.1±5.9 |
| Women | Age -39 | 22.7±3.6 | 19.7±7.2 | 37.9±4.2 | 35.7±3.9 | 25.4±4.2 |
|  | Age 40-59 | 22.6±3.7 | 18.9±6.4 | 37.5±3.6 | 35.4±3.3 | 25.3±3.9 |
|  | Age 60- | 23.3±3.7 | 18.7±6.4 | 35.7±3.6 | 33.8±3.3 | 23.4±3.9 |

BMI: body mass index

Table S2. Adjusted odds ratios for having DM or pre-DM for 5kg increase in handgrip strength

|  | Model | N | aOR (95% CI) |
| --- | --- | --- | --- |
| FFM model 1 | | 1940 | **0.810 (0.729-0.899)** |
|  | BMI <18.5 | 97 | **0.511 (0.276-0.948)** |
|  | BMI 18.5-24.9 | 1181 | 0.929 (0.805-1.072) |
|  | BMI ≥25.0 | 662 | **0.769** **(0.650-0.910)** |
| BMI model 1 | | 1940 | **0.848 (0.708-0.937)** |
|  | BMI <18.5 | 97 | 0.717 (0.424-1.212) |
|  | BMI 18.5-24.9 | 1181 | 0.913 (0.799-1.042) |
|  | BMI ≥25.0 | 662 | **0.805** **(0.685-0.946)** |

aOR: adjusted odds ratio; 95%CI: 95% confidence interval

FFM: fat free mass; BMI: body mass index
